# Unveiling hidden diversity: new records of *Chaitophorus* (Hemiptera, Aphididae) in Korea from historical specimens

**DOI:** 10.3897/BDJ.13.e150852

**Published:** 2025-05-07

**Authors:** Hwalran Choi, Yeyeun Kim, Seunghwan Lee

**Affiliations:** 1 Insect Biosystematics Laboratory, Research Institute of Agricultural and Life Sciences, Department of Agricultural Bio-technology, Seoul National University, Seoul, Republic of Korea Insect Biosystematics Laboratory, Research Institute of Agricultural and Life Sciences, Department of Agricultural Bio-technology, Seoul National University Seoul Republic of Korea

**Keywords:** aphids, Salicaceae, East Asia

## Abstract

**Background:**

The aphid genus *Chaitophorus* Koch, 1854 (Hemiptera, Aphididae, Chaitophorinae) has been studied in Korea from historical specimens.

**New information:**

Korean *Chaitophorus* aphids have been confirmed, including four new records: *C.horii* Takahashi, 1939; *C.leucomelas* Koch, 1854; *C.salijaponicus* Essig and Kuwana, 1918; and *C.tremulae* Koch, 1854. These four newly-recorded species are described with biometric measurements and illustrations. A modified taxonomic key for Korean *Chaitophorus* spp. is presented.

## Introduction

The aphid genus *Chaitophorus* Koch, 1854 is a species-rich group within the subfamily Chaitophorinae. These aphids are distributed across the Holarctic Region, with approximately 90 species recorded (Blackman and Eastop 2024, Favret and Aphid Taxon Community, eds. 2025). *Chaitophorus* species are well-known for their tree-dwelling habit, with no host alternation and they exhibit host specificity at the genus level, feeding exclusively on either *Salix* or *Populus* (Salicaceae), but never both (Blackman and Eastop 2024). Distinct morphological traits of the genus include a body covered with long setae, short siphunculi and knobbed cauda, all of which are particularly useful for distinguishing these aphids from related groups.

Taxonomic studies on *Chaitophorus* have been conducted in various countries. Pintera (1987) revised the Palaearctic fauna, covering 58 species. Additionally, research has been conducted in China and Japan by Tao (1964) and Higuchi (1972), respectively. Nieto Nafría and Mier Durante (1998) and Wieczorek and Osiadacz (2007) reviewed the European species, while Chakrabarti (1977) and Ghosh (1980) studied the species found in India. In North America, Hille Ris Lambers (1960) and Richards (1972) conducted reviews.

Systematic and co-evolutionary studies on the genus *Chaitophorus* have also been undertaken. Shingleton and Stern (2003) were the first to conduct a molecular phylogenetic study on this genus, using mitochondrial DNA (mtDNA) from 15 species. Wieczorek and Kajtoch (2011) and Wieczorek et al. (2017) examined the tribal relationships within Chaitophorinae and their closely-related groups using both mtDNA and nuclear genes. Yang et al. (2016) investigated the co-evolution of *Chaitophorus* with the symbiont *Buchnera* and Liu et al. (2022) explored macroevolution in relation to host plants.

Taxonomic research on Korean *Chaitophorus* species has been conducted by several researchers, including Shinji (1941), Paik (1972), Szelegiewicz (1981), Pintera (1987) and Lee and Seo (1992). In this study, we newly record four species of *Chaitophorus* collected from Korea.

## Materials and methods

Historical specimens of this study are deposited in the Consortium of Korea Biodiversity and Sustainable Use, College for Agriculture and Life Sciences, Seoul National University (CALS SNU, Korea).

Illustrations for each species were taken by digital camera (14.2 Color Mosaic, Diagnostic Instruments, Sterling Heights, MI, USA) attached to the microscope (DM 400B, Leica Microsystems, Wetzlar, Germany) at a resolution of 600 dpi. Measurements for each specimen are taken from the digital images by Image Laboratory v.2.2.4.0 software (MCM Design Ltd, Hillerod, Denmark).

Abbreviations used for descriptions are as follows: ap., apterous viviparous female; Ant., antennae; Ant. I, Ant. II, Ant. III, Ant. IV, Ant. V, Ant. VI and Ant. VIb, antennal segments I, II, III, IV, V, VI and base of VI, respectively; BDAnt. III, basal diameter of antennal segment III; AbdT. III, Abdominal Tergite III; GP, genital plate; 2HT, second segment of hind tarsus; PT, processus terminals of antennal segment VI; SIPH, siphunculus; URS, ultimate rostral segments (segment IV+V). For the localities of distribution, provincial abbreviations are also used: South Korea: CB, Chungcheongbuk-do; GG, Gyeonggi-do; JB, Jeollabuk-do; North Korea: HB, Hwanghaebuk-do; PB, Pyeonganbuk-do; PN: Pyeongannam-do; RG: Ryanggang-do.

## Taxon treatments

### Chaitophorus horii

Takahashi, 1939

2FBFA0B5-C73A-54A6-A0BB-4B39D83F80A1

#### Materials

- **Type status:** Other material. **Occurrence:** recordedBy: J. Havelka; individualCount: 3; sex: female; lifeStage: adult; occurrenceStatus: present; disposition: in collection; associatedOccurrences: host: *Salixherbacea*; occurrenceID: 19819F5C-4912-5340-A02F-DFD62934B644; **Taxon:** taxonID: 2074388; namePublishedInID: http://hdl.handle.net/2115/9419; scientificName: *Chaitophorushorii* Takahashi, 1939; namePublishedIn: Takahashi, R. (1939) Some Aphididae from Hokkaido (Hemiptera). Insecta Matsumurana, 13(4), 114–128.; kingdom: Animalia; phylum: Arthropoda; class: Insecta; order: Hemiptera; family: Aphididae; genus: Chaitophorus ; specificEpithet: horii; nomenclaturalCode: ICZN; taxonomicStatus: accepted; **Location:** continent: Asia; country: North Korea; countryCode: KP; stateProvince: Ryanggang Province; municipality: Samjiyeon; locality: Mt. Baekdu; decimalLatitude: 41.899; decimalLongitude: 128.394; geodeticDatum: WGS84; coordinateUncertaintyInMeters: 100; **Identification:** identifiedBy: Yeyeun Kim; dateIdentified: 2025-02-18; **Event:** samplingProtocol: hand collected; eventDate: 1988-06-24; year: 1988; month: 6; day: 24; **Record Level:** type: specimen; modified: 2025-03-28; language: en; rights: Seoul National University; rightsHolder: Content licensed under Seoul National University; accessRights: not for profit use only; institutionID: NPRI; collectionID: SNU; basisOfRecord: PreservedSpecimen
- **Type status:** Other material. **Occurrence:** recordedBy: J. Havelka; individualCount: 2; sex: female; lifeStage: adult; occurrenceStatus: present; disposition: in collection; associatedOccurrences: host: *Salixherbacea*; occurrenceID: F12B8F44-2F65-551B-8C05-8C7EE953F41E; **Taxon:** taxonID: 2074388; namePublishedInID: http://hdl.handle.net/2115/9419; scientificName: *Chaitophorushorii* Takahashi, 1939; namePublishedIn: Takahashi, R. (1939) Some Aphididae from Hokkaido (Hemiptera). Insecta Matsumurana, 13(4), 114–128.; kingdom: Animalia; phylum: Arthropoda; class: Insecta; order: Hemiptera; family: Aphididae; genus: Chaitophorus ; specificEpithet: horii; nomenclaturalCode: ICZN; taxonomicStatus: accepted; **Location:** continent: Asia; country: North Korea; countryCode: KP; stateProvince: Ryanggang Province; municipality: Samjiyeon; locality: Mt. Baekdu; decimalLatitude: 41.899; decimalLongitude: 128.394; geodeticDatum: WGS84; coordinateUncertaintyInMeters: 100; **Identification:** identifiedBy: Yeyeun Kim; dateIdentified: 2025-02-18; **Event:** samplingProtocol: hand collected; eventDate: 1988-06-17; year: 1988; month: 6; day: 17; **Record Level:** type: specimen; modified: 2025-03-28; language: en; rights: Seoul National University; rightsHolder: Content licensed under Seoul National University; accessRights: not for profit use only; institutionID: NPRI; collectionID: SNU; basisOfRecord: PreservedSpecimen

#### Description

**Figure 1.**
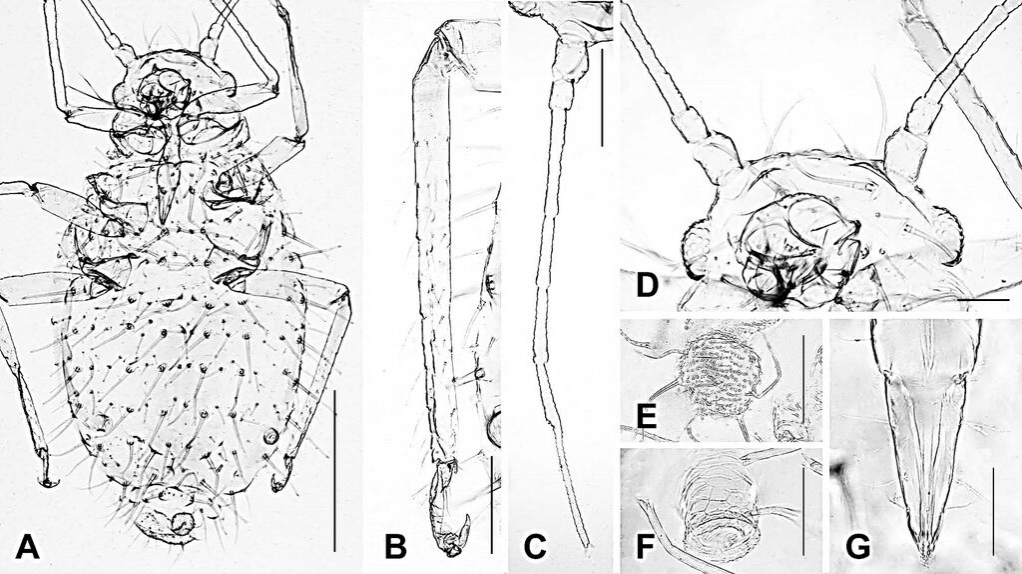
Apterous viviparous female of *Chaitophorushorii* Takahashi, 1939. **A** whole body; **B** hind tibia and tarsus; **C** antenna; **D** head; **E** cauda; **F** siphunculus; **G** ultimate rostral segments. Scale bar: A, 0.5 mm; B,C, 0.1 mm; D, 0.1 mm; E,F,G, 0.05 mm.

**Apterous viviparous female** (Fig. 1, Table 1). **Colour (macerated specimens).** Body and appendages wholly pale. **Morphology.** Body elongated and oval-shaped; its length twice as long as width, about 1.36 mm. **Head**: length of antenna slightly over 0.5 times of the body length; PT almost 2 times longer than Ant. VIb.; longest seta on Ant. III same as long as basal width of segment. URS beak-shaped, with 6-7 setae; nearly 1.1 times as long as 2HT. **Thorax**: Legs comparatively shorter than other *Chaitophorus* spp., hind femur and tibia about 0.29 and 0.45 mm long, respectively. **Abdomen**: abdominal segments fused from I to VII; most setae on tergite relatively long, stunted or furcate; nearly 10 setae each on tergite VI and VIII. SIPH slightly long and stump-shaped. Cauda knobbed with 5-6 setae.

##### Host plants

*Salixcaprea*, *S.herbacea*, *S.rorida*, *S.viminalis*, *Salix* spp. (Blackman and Eastop 2024).

#### Diagnosis

*Chaitophorushorii* morphologically resembles *C.purpureae*. However, it is distinguished by its beak-shaped URS and long dorsal setae.

#### Distribution

Korea (new record), South Europe, Japan, Thailand (Blackman and Eastop 2024).

### Chaitophorus leucomelas

Koch, 1854

81819250-9F6F-534D-9A7D-4296375FAE83

#### Materials

- **Type status:** Other material. **Occurrence:** catalogNumber: 87HA1644; recordedBy: J. Havelka; individualCount: 24; sex: females; lifeStage: adult; occurrenceStatus: present; disposition: in collection; associatedOccurrences: host: *Populusalba*; occurrenceID: 71388D05-EB1D-5BF5-A4D7-514768BE84CB; **Taxon:** taxonID: 2074434; namePublishedInID: https://doi.org/10.5281/zenodo.14387414; scientificName: *Chaitophorusleucomelas* Koch, 1854; namePublishedIn: Koch, C.L. (1854) In *Die Pflanzenläuse Aphiden getreu nach dem Leben abgebildet und beschrieben*. J.L. Lotzbeck, Nürnberg. Vol. 1, 1–36 pp.; higherClassification: Animalia; kingdom: Animalia; phylum: Arthropod; class: Insects; order: Hemiptera; family: Aphididae; genus: Chaitophorus; specificEpithet: *leucomelas*; taxonRank: species; scientificNameAuthorship: Koch, 1854; nomenclaturalCode: ICZN; taxonomicStatus: accepted; **Location:** continent: Asia; country: North Korea; countryCode: KP; stateProvince: South Hwanghae; municipality: Kaesong; locality: Gongmin Wang Tomb; decimalLatitude: 37.982; decimalLongitude: 126.473; geodeticDatum: WGS84; coordinateUncertaintyInMeters: 100; **Identification:** identifiedBy: Yeyeun Kim; dateIdentified: 2025-03-28; **Event:** samplingProtocol: hand collected; eventDate: 1987-06-01; year: 1987; month: 06; day: 01; **Record Level:** type: specimen; modified: 2025-03-28; language: en; rights: Seoul National University; rightsHolder: Seoul National University; accessRights: not for profit use only; institutionID: NPRI; collectionID: SNU
- **Type status:** Other material. **Occurrence:** catalogNumber: #5783; recordedBy: J. Havelka; individualCount: 4; sex: females; lifeStage: adult; occurrenceStatus: present; disposition: in collection; associatedOccurrences: host: *Populus* sp.; occurrenceID: D29D7CDD-DF15-5196-87CE-EEE7A14FDD6F; **Taxon:** taxonID: 2074434; namePublishedInID: https://doi.org/10.5281/zenodo.14387414; scientificName: *Chaitophorusleucomelas* Koch, 1854; namePublishedIn: Koch, C.L. (1854) In *Die Pflanzenläuse Aphiden getreu nach dem Leben abgebildet und beschrieben*. J.L. Lotzbeck, Nürnberg. Vol. 1, 1–36 pp.; higherClassification: Animalia; kingdom: Animalia; phylum: Arthropod; class: Insects; order: Hemiptera; family: Aphididae; genus: Chaitophorus ; specificEpithet: *leucomelas*; taxonRank: species; scientificNameAuthorship: Koch, 1854; nomenclaturalCode: ICZN; taxonomicStatus: accepted; **Location:** continent: Asia; country: South Korea; countryCode: KR; stateProvince: North Jeolla Province; municipality: Muju; decimalLatitude: 36.019; decimalLongitude: 127.658; geodeticDatum: WGS84; coordinateUncertaintyInMeters: 100; **Identification:** identifiedBy: Yeyeun Kim; dateIdentified: 1989-07-10; **Event:** samplingProtocol: direct collection; eventDate: 1969-10-29; year: 1969; month: 10; day: 29; **Record Level:** type: specimen; modified: 1969-10-29; language: en; rights: Seoul National University; rightsHolder: Seoul National University; accessRights: not for profit use only; institutionID: NPRI; collectionID: #5783; basisOfRecord: PreservedSpecimen

#### Description

**Figure 2.**
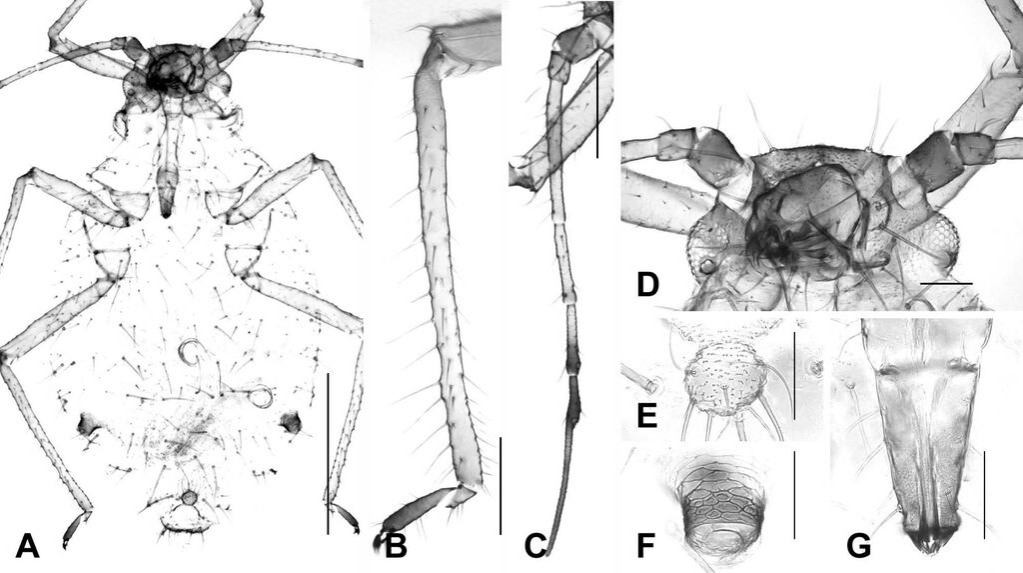
Apterous viviparous female of *Chaitophorusleucomelas* Koch, 1854. **A** whole body; **B** hind tibia and tarsus; **C** antenna; **D** head; **E** cauda; **F** siphunculus; **G** ultimate rostral segments. Scale bar: A, 0.5 mm; B,C, 0.1 mm; D, 0.1 mm; E,F,G, 0.05 mm.

**Apterous viviparous female** (Fig. 2, Table 2). **Colour** (macerated specimens). Body pale, leg, cauda and antenna faintly grey. Head, mandibular lamina, whole Ant. except Ant. III through apical half of Ant. V and SIPH more deeply pigmented. URS dark in peak. **Morphology.** Body ovoid, about 1.98 mm long. **Head**: flat with stout seta on frons; mandibular lamina with eight or more setae. Antennal length almost 0.6 times as long as body length; PT almost 3 times longer than Ant. VIb. URS short with eight or more setae. **Thorax**: pronotum smooth. Hind femur and tibia length about 0.51 mm and 0.69 mm, respectively; hind tibia entirely with long and fine setae. **Abdomen**: abdominal segments fused from II to VII; dorsal cuticles with nodules and faintly pigmented, with fine, long and pointed setae. SIPH 0.02 times the body length. Cauda knobbed with eight setae.

##### Host plants

*Populusalba*, *P.deltoides*, *P.nigra*, *P.simonii*, *Populus* spp. (Blackman and Eastop 2024).

#### Diagnosis

This species typically has pigmentation on the dorsum, frons and SIPH, but the pigmentation on the dorsum varies depending on the specimen, whereas the pigmentation on the frons and SIPH is always present. Korean *Chaitophorusleucomelas* has faintly pigmented dorsum, but the frons and SIPH distinctly have pigmentation. Additionally, the pigmentation of antennae and their ratio and dorsal cuticles with nodules follow the referred description (Pintera 1987).

#### Distribution

Korea (new record), China, Kazakhstan, Mongolia, Siberia, Europe, Canada, USA, Chile, Africa (Blackman and Eastop 2024).

### Chaitophorus salijaponicus

Essig and Kuwana, 1918

8DFC74BB-C604-5442-9944-B929AA61EB85

#### Materials

- **Type status:** Other material. **Occurrence:** catalogNumber: #88HA3688; recordedBy: J. Havelka; individualCount: 8; sex: females; lifeStage: adult; occurrenceStatus: present; disposition: in collection; associatedOccurrences: host: *Salixpurpurea*; occurrenceID: C7336A4E-0FF3-5014-B231-3CA2EEC96C4B; **Taxon:** taxonID: 2074456; namePublishedInID: 10.1163/187631204788920158; scientificName: *Chaitophorussalijaponicus* Essig and Kuwana, 1918; namePublishedIn: Essig, E.O. & Kuwana, S.I. (1918) Some Japanese Aphididae. *Proceedings of the California Academy of Sciences*, 8(3), 35–112.; higherClassification: Animalia; kingdom: Animalia; phylum: Arthropoda; class: Insecta; order: Hemiptera; family: Aphididae; genus: Chaitophorus; specificEpithet: *salijaponicus*; taxonRank: species; nomenclaturalCode: ICZN; **Location:** continent: Asia; country: North Korea; countryCode: KP; stateProvince: Ryanggang Province; locality: Hyesan; decimalLatitude: 41.387; decimalLongitude: 128.173; geodeticDatum: WGS84; coordinateUncertaintyInMeters: 100; **Identification:** identifiedBy: Yeyeun Kim; dateIdentified: 2025-03-28; **Event:** samplingProtocol: hand collected; eventDate: 1988-06-28; year: 1988; month: 06; day: 28; **Record Level:** type: specimen; modified: 2025-03-28; language: en; rights: Seoul National University; rightsHolder: Seoul National University; accessRights: not for profit use only; institutionID: NPRI; collectionID: SNU
- **Type status:** Other material. **Occurrence:** catalogNumber: #5718; recordedBy: J. Havelka; individualCount: 2; sex: females; lifeStage: adult; occurrenceStatus: present; disposition: in collection; associatedOccurrences: host: *Salix* sp.; occurrenceID: 4A417E0F-2855-5528-B85B-9E47C1522690; **Taxon:** taxonID: 2074456; namePublishedInID: 10.1163/187631204788920158; scientificName: *Chaitophorussalijaponicus* Essig and Kuwana, 1918; namePublishedIn: Essig, E.O. & Kuwana, S.I. (1918) Some Japanese Aphididae. *Proceedings of the California Academy of Sciences*, 8(3), 35–112.; higherClassification: Animalia; kingdom: Animalia; phylum: Arthropoda; class: Insecta; order: Hemiptera; family: Aphididae; genus: Chaitophorus; specificEpithet: *salijaponicus*; taxonRank: species; nomenclaturalCode: ICZN; **Location:** continent: Asia; country: South Korea; countryCode: KR; stateProvince: North Jeolla Province; municipality: Muju; decimalLatitude: 36.014; decimalLongitude: 127.656; geodeticDatum: WGS84; coordinateUncertaintyInMeters: 100; **Identification:** identificationID: Yeyeun Kim; identifiedBy: Yeyeun Kim; dateIdentified: 2025-02-19; **Event:** samplingProtocol: hand collected; eventDate: 1969-10-29; year: 1969; month: 10; day: 29; **Record Level:** type: specimen; modified: 2025-03-28; language: en; rights: Seoul National University; rightsHolder: Seoul National University; accessRights: not for profit use only; institutionID: SNU; collectionID: #5718

#### Description

**Figure 3.**
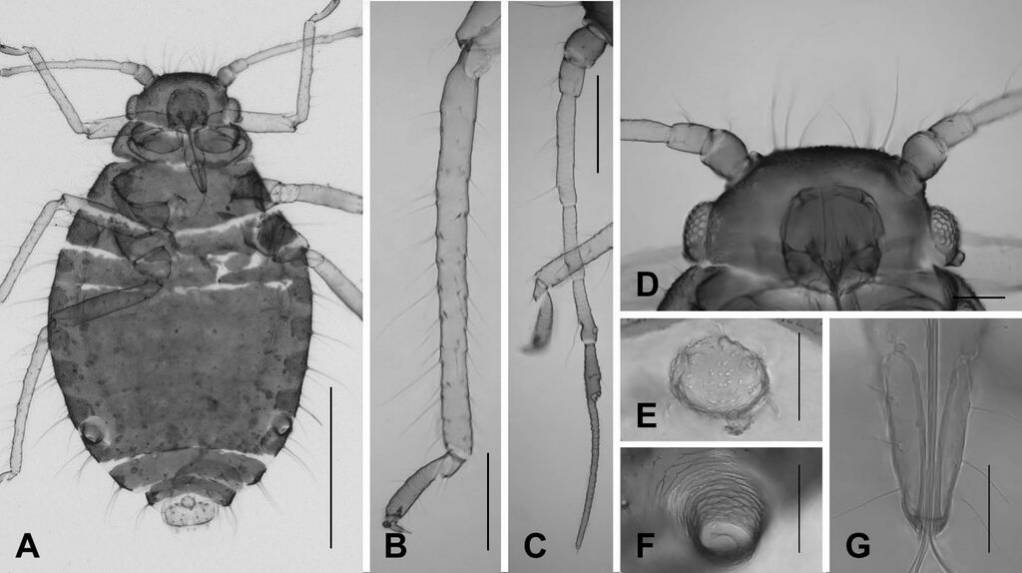
Apterous viviparous female of *Chaitophorussalijaponicus* Essig and Kuwana, 1918. **A** whole body; **B** hind tibia and tarsus; **C** antenna; **D** head; **E** cauda; **F** siphunculus; **G** ultimate rostral segments. Scale bar: A, 0.5 mm; B,C, 0.1 mm; D, 0.1 mm; E,F,G, 0.05 mm.

**Apterous viviparous female** (Fig. 3, Table 3). **Colour** (macerated specimens). Body blackish and oval-shaped, intersegmentally pale on unfused tergites. Antenna and leg slightly grey; hind tibia, Ant. VIb and PT darker than body. Abdominal tergite with a pale ring around base of SIPH. **Morphology.** Body elongated oval-shaped; about 1.52 mm long; marginal setae around body distinctly long and fine. **Head**: flat with long pointed setae on frons; mandibular lamina with eight setae. Antenna slender; whole antennae almost 0.55 times as long as body length; PT 2.55 times as long as Ant. VIb.; Ant. III with seven long setae; Ant. V with relatively stunt setae. URS with eight setae. **Thorax**: hind femur and tibia about 0.31 mm and 0.49 mm long, respectively; length of leg a little shorter than other species in comparison with length of body. **Abdomen**: abdominal tergites from II to VII fused; more sclerotised on marginal tergites of each segment. SIPH short and reticulate. Cauda knobbed with 6 setae.

##### Host plants

*S.babylonica*, *S.caprea*, *S.integra*, *S.koriyanagi*, *Salix* spp. (Blackman and Eastop 2024).

#### Diagnosis

*C.salijaponicus* closely resembles *C.hokkaidensis* morphologically. However, it can be distinguished by the state of abdominal tergite I: in *C.salijaponicus*, it is free, whereas in *C.hokkaidensis*, it is completely fused with the preceding segment.

#### Distribution

Korea (new record), China, Siberia, Mongolia, Japan (Blackman and Eastop 2024).

### Chaitophorus tremulae

Koch, 1854

D74538B2-97A7-54B1-B98B-706380E608C8

#### Materials

- **Type status:** Other material. **Occurrence:** catalogNumber: #88HA3711; recordedBy: J. Havelka; individualCount: 6; sex: female; lifeStage: adult; occurrenceStatus: present; disposition: in collection; associatedOccurrences: host: *Populusdavidiana*; occurrenceID: 045CC865-6020-5684-B585-D80F05169086; **Taxon:** taxonID: 2074419; namePublishedInID: 10.5281/zenodo.14387432; scientificName: *Chaitophorustremulae* Koch, 1854; namePublishedIn: Koch, C.L. (1854) In *Die Pflanzenläuse Aphiden getreu nach dem Leben abgebildet und beschrieben*. J.L. Lotzbeck, Nürnberg. Vol. 1, 1–36 pp.; higherClassification: Animalia; kingdom: Animalia; phylum: Arthropod; class: Insects; order: Hemiptera; family: Aphididae; genus: Chaitophorus; specificEpithet: *tremulae*; taxonRank: species; nomenclaturalCode: ICZN; **Location:** continent: Asia; country: North Korea; countryCode: KP; stateProvince: Ryanggang Province; municipality: Hyesan; decimalLatitude: 41.387; decimalLongitude: 128.174; geodeticDatum: WGS84; coordinateUncertaintyInMeters: 100; **Identification:** identifiedBy: yeyeun Kim; dateIdentified: 2025-02-19; **Event:** samplingProtocol: hand collected; eventDate: 1988-06-28; year: 1988; month: 06; day: 28; **Record Level:** type: specimen; modified: 2025-03-28; language: en; rights: Seoul National University; rightsHolder: Seoul National University; accessRights: not for profit use only; institutionID: NPRI; collectionID: SNU; basisOfRecord: PreservedSpecimen
- **Type status:** Other material. **Occurrence:** catalogNumber: #5895; recordedBy: J. Havelka; individualCount: 2; sex: female; lifeStage: adult; occurrenceStatus: present; disposition: in collection; associatedOccurrences: host: *Populusdavidiana*; occurrenceID: B1911DEB-ECE7-5D4C-B3C1-B6F695873087; **Taxon:** taxonID: 2074419; namePublishedInID: 10.5281/zenodo.14387432; scientificName: *Chaitophorustremulae* Koch, 1854; namePublishedIn: Koch, C.L. (1854) In *Die Pflanzenläuse Aphiden getreu nach dem Leben abgebildet und beschrieben*. J.L. Lotzbeck, Nürnberg. Vol. 1, 1–36 pp.; higherClassification: Animalia; kingdom: Animalia; phylum: Arthropod; class: Insects; order: Hemiptera; family: Aphididae; genus: Chaitophorus; specificEpithet: *tremulae*; taxonRank: species; nomenclaturalCode: ICZN; taxonomicStatus: accepted; **Location:** continent: Asia; country: South Korea; countryCode: KR; municipality: Seoul; decimalLatitude: 37.475; decimalLongitude: 126.948; geodeticDatum: WGS84; coordinateUncertaintyInMeters: 100; **Identification:** identifiedBy: yeyeun Kim; dateIdentified: 2025-02-19; **Event:** samplingProtocol: hand collected; eventDate: 1970-05-06; year: 1970; month: 05; day: 06; **Record Level:** type: specimen; modified: 2025-03-28; language: en; rights: Seoul National University; rightsHolder: Seoul National University; accessRights: not for profit use only; institutionID: NPRI; collectionID: SNU; basisOfRecord: PreservedSpecimen

#### Description

**Figure 4.**
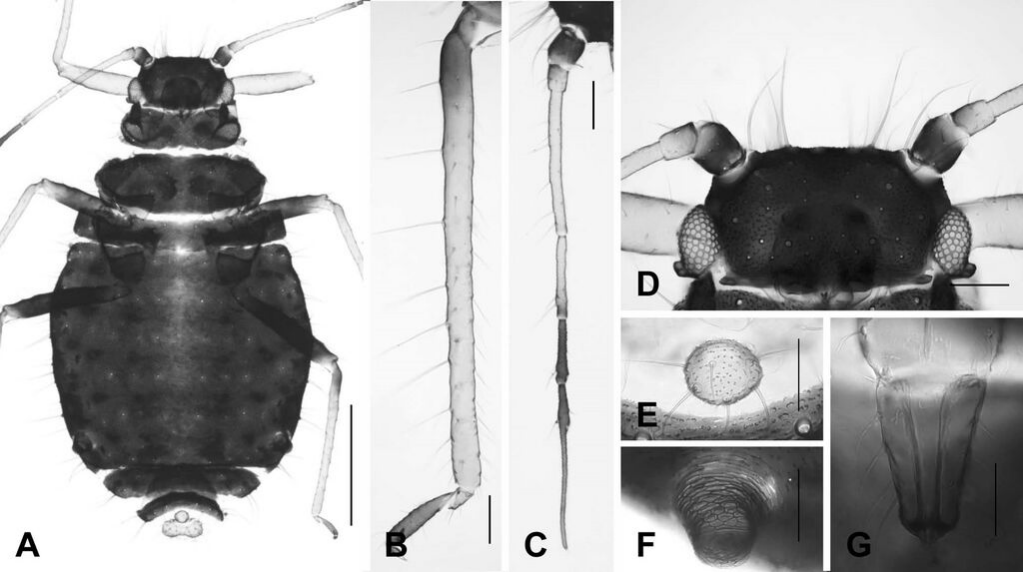
Apterous viviparous female of *Chaitophorustremulae* Koch, 1854. **A** whole body; **B** hind tibia and tarsus; **C** antenna; **D** head; **E** cauda; **F** siphunculus; **G** ultimate rostral segments. Scale bar: A, 0.5 mm; B,C, 0.1 mm; D, 0.1 mm; E,F,G, 0.05 mm.

**Apterous viviparous female** (Fig. 4, Table 4). **Colour** (macerated specimens). Body extremely blackish, slightly bright in the spinal part of abdomen. Antennae pale, except Ant. I, distal part of Ant. IV, Ant. V., Ant. VIb and PT. Legs pale, except mid- and hind femora, basal part of hind tibia and 2HT. Cauda and anal plate distinctly pale. SIPH dark as body colour. **Morphology.** Body elongated oval-shaped; about 2.20 mm long. **Head**: smooth, seta very long and spine. Antenna slender; whole antennae almost 0.57 times as long as body length; PT 2.73 times as long as Ant. VIb. URS short and slightly blunt, with eight or more setae. **Thorax**: pronotum smooth. Hind femur and tibia about 0.55 mm and 0.69 mm long, respectively; **Abdomen**: abdominal tergites fused from I to VI, with blunt or furcate seta; dorsum with pore-like microstructure in a row of each segment. SIPH short and finely reticulate. Cauda distinctly knobbed with six setae.

##### Host plants

*P.maximowiczii*, *P.sieboldii*, *P.simonii*, *P.tremula* (*Blackman and Eastop 2024*).

#### Diagnosis

*C.tremulae* closely resembles *C.salijaponicus* morphologically. However, it can be distinguished by body length and marginal tergites: in *C.tremulae*, body length is longer than 1.8 mm and has no sclerotised marginal tergites, whereas in *C.salijaponicus*, body length is shorter than 1.8 mm and sclerotised marginal tergites are developed.

#### Distribution

Korea (new record), Great Britain, Netherlands, Germany, Denmark, Spain, Norway, Finland, Poland, Hungary, Turkey, Russia, Caucasus, Kazakhstan, Siberia, China, Mongolia, Japan (Blackman and Eastop 2024).

## Identification Keys

### Key to the species of the genus *Chaitophorus* in the Korean Peninsula (apterous viviparous female) (modified from Albert Pintera (1987) and Blackman and Eastop (2024)).

**Table:** 

1	Pseudosensoria present on hind tibia	2
–	Pseudosensoria absent on hind tibia	3
2	Pseudosensoria scattered over nearly whole hind tibia. Live on *Populus* spp.	***C.populeti*** **(Panzer, 1801)**
–	Pseudosensoria (approximately 12) on the enlarged part near the knees of the hind tibia. Live on *Salix* spp.	***C.saliniger* Shinji, 1924**
3	Body wholly pale	4
–	Body wholly or partly pigmented	11
4	Abdominal tergite I fused with the following tergite. URS with beak-shaped or U-shaped	5
–	Abdominal tergite I not fused with the following tergite. URS with U-shaped	6
5	URS with beak-shaped. URS/2HT 1.1. PT/Ant. VIb 2.0. Dorsal setae usually furcated. Live on *Salix* spp.	***C.horii* Takahashi, 1939**
–	URS with U-shaped. URS/2HT 0.8. PT/Ant. VIb 2.0-2.5. Dorsal setae long, not furcated. Live on Salixpurpureavar.smithiana	***C.purpureae* Lee and Seo, 1992**
6	Abdominal tergite VII completely fused with the preceding tergite. SIPH dark. Live on *Populus* spp.	***C.leucomelas* Koch, 1854**
–	Abdominal tergite VII mostly free with the preceding tergite. SIPH pale	7
7	Longest seta on Ant. III and AbdT. III almost 3-6 times as long as the basal diameter of Ant. III, respectively. Live on *Populus* spp.	***C.populialbae* (Boyer de Fonscolombe, 1841)**
–	Longest seta on Ant. III and AbdT. III less than 1.2 times and 3.2 times as long as the basal diameter of Ant. III, respectively	8
8	Body elongate, oval without pigmentation. Microstructure consisting of blunt nodules, very sparsely distributed in the middle of dorsum and head, often poorly visible. Live on *Salix* spp.	***C.saliapterus* Shinji, 1924**
–	Body rather elongate, oval and fuscous. Microstructure not developed or, at most, with small granules on the frons. Live on *Salix* spp. or *Populus* spp.	9
9	Seta on Ant. III short, not more than twice as long as basal diameter of Ant. III. Ant. V with 2-3 setae. SIPH pigmented and reticulated on all their length. Live on *Salixbakko*	***C.matsumurai* Hille Ris Lambers, 1960**
–	Seta on Ant. III more than 4 times as long as basal diameter of Ant. III. PT more than 4 times as long as basal part of Ant. VI. Microstructure developed only with small granules on the frons. Live on *Populuskoreanus*	***C.variegatus* Szelegiewicz, 1981**
10	Body length longer than 1.8 mm, usually over 2 mm. Live on *Populus* spp.	***C.tremulae* Koch, 1854**
–	Body length 1.3-1.9 mm. Live on *Salix* spp.	11
11	Abdominal tergite I free. Body length shorter than 1.8 mm, usually about 1.5 mm. Live on *Salix* spp.	***C.salijaponicus* Essig and Kuwana, 1918**
–	Abdominal tergite I completely fused with the preceding one. Body length shorter than 1.8 mm, usually about 1.4 mm. Live on *Salix* spp.	***C.hokkaidensis* Higuchi, 1972**

## Discussion

**Figure 5.**
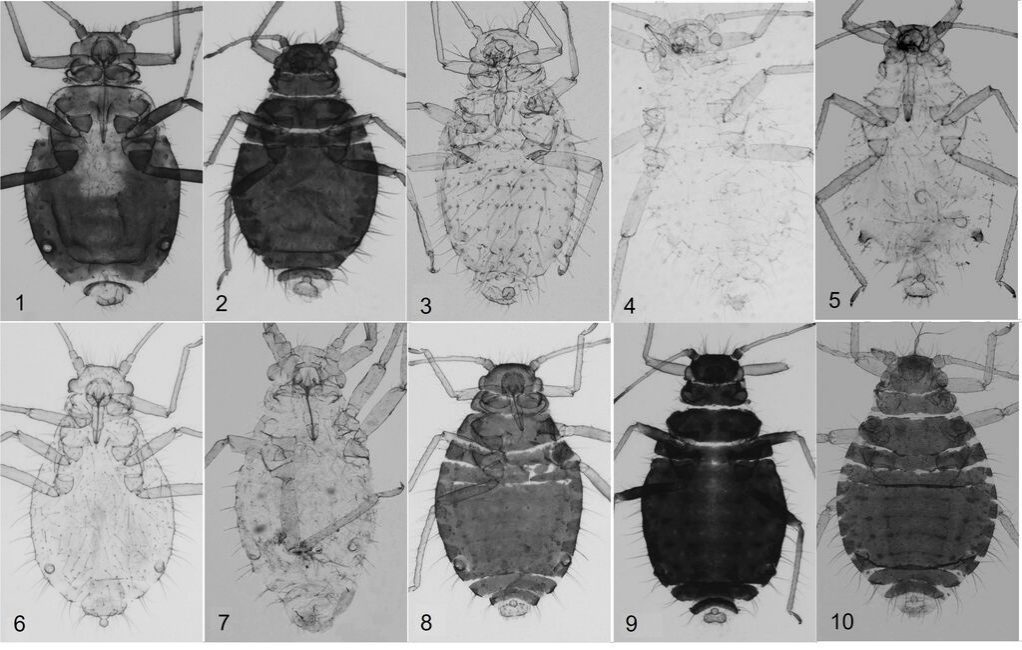
Apterous viviparous females of Korean *Chaitophorus* spp. from this study. 1. *C.populeti* 2. *C.saliniger* 3. *C.horii* 4. *C.purpureae* 5. *C.leucomelas* 6. *C.populialbae* 7. *C.saliapterus* 8. *C.salijaponicus* 9. *C.tremulae* 10. *C.hokkaidensis*

*Chaitophorus* species have been studied from 311 historically old slide-mounted specimens collected across North and South Korea and stored at SNU. Amongst these, we confirmed 10 valid species: *C.horii* Takahashi, 1939 (new record); *C.leucomelas* Koch, 1854 (new record); *C.salijaponicus* Essig and Kuwana, 1918 (new record); *C.tremulae* Koch, 1854 (new record); *C.hokkaidensis* Higuchi, 1972; *C.populeti* (Panzer, 1801); *C.populialbae* (Boyer de Fonscolombe, 1841); *C.purpureae* Lee and Seo, 1992; *C.saliapterus* Shinji, 1924; and *C.saliniger* Shinji, 1924 (Fig. 5).

In 1941, Shinji reported *C.narae* Shinji, 1941 for the first time from a Korean specimen. However, this species was associated with a host plant from the order Fagales — an atypical host for *Chaitophorus*, which primarily feeds on *Salix* and *Populus* species. Additionally, Shinji’s morphological description of the species lacked sufficient detail, complicating subsequent taxonomic efforts. The combination of an unusual host plant, the limited number of specimens and inadequate morphological characterisation has hindered further research on this species. As a result, *C.narae* Shinji, 1941 has been excluded from the taxonomic key of Korean *Chaitophorus* species.

*C.variegatus* and *C.matsumurai* were recorded by Szelegiewicz (1981) and Paik (1972), respectively (with the original record of *C.salicicolus*). However, due to a lack of specimens and the absence of any subsequent records, further confirmation of these species is required.

For future studies, an integrated classification system, based on morphological characteristics and DNA barcode data is necessary, along with a comparative analysis with global datasets.

## Supplementary Material

XML Treatment for Chaitophorus horii

XML Treatment for Chaitophorus leucomelas

XML Treatment for Chaitophorus salijaponicus

XML Treatment for Chaitophorus tremulae

## Figures and Tables

**Table 1.** Biometric data of *Chaitophorushorii*.

Part		Apterous vivipara (n = 5)	
		Average	(Min-Max)
Length (mm)	Body (from the most forward point of the frons to end of Cauda)	1.36	(1.35-1.38)
	Whole Ant.	0.71	(0.69-0.72)
	Ant. I	0.05	(0.05-0.05)
	Ant. II	0.04	(0.04-0.04)
	Ant. III	0.14	(0.13-0.14)
	Ant. IV	0.10	(0.09-0.10)
	Ant. V	0.10	(0.10-0.11)
	Ant. VIb	0.08	(0.08-0.08)
	PT	0.17	(0.16-0.17)
	URS	0.09	(0.09-0.10)
	Hind femur	0.29	(0.29-0.30)
	Hind tibia	0.45	(0.45-0.47)
	2HT	0.09	(0.08-0.09)
	SIPH	0.02	(0.02-0.03)
	Knob of Cauda	0.05	(0.05-0.05)
	Width of the knob of Cauda	0.05	(0.05-0.05)
	Longest seta on Ant. III	0.02	(0.01-0.02)
	Longest seta on AbdT. III	0.14	(0.14-0.15)
No. of setae on	Mandibular lamina	6	(6-7)
	Ant. I	3	(3-4)
	Ant. II	3	(3-3)
	Ant. III	2	(2-3)
	URS (subsidiary)	6	(6-7)
	Tergite VI between SIPH	10	(9-11)
	Tergite VIII	11	(10-11)
	Median of GP	4	(3-6)
	Posterior margin of GP	13	(12-15)
	Knob of Cauda	7	(6-8)
No. of secondary rhinaria on	Ant. III	0	
	Ant. IV	0	
	Ant. V	0	
Ratio (times)	Whole antennae / Body	0.52	(0.51-0.53)
	PT / Ant. VIb	1.97	(1.94-1.97)
	PT / Ant. III	1.21	(1.21-1.21)
	URS / 2HT	1.10	(1.10-1.11)
	URS / Ant.VIb	1.14	(1.14-1.15)
	SIPH / Body	0.02	(0.02-0.02)
	SIPH / Ant. III	0.21	(0.20-0.21)
	SIPH / Hind femur	0.10	(0.10-0.10)
	SIPH / Cauda	0.49	(0.47-0.51)
	Knob of Cauda / Width of the knob of Cauda	1.00	(1.00-1.02)
	Seta on Ant. III / Ant. IIIBD	1.00	(0.90-1.00)
	Seta on AbdT. III / Ant. IIIBD	7.35	(7.14-7.37)

**Table 2.** Biometric data of *Chaitophorusleucomelas*.

Part		Apterous vivipara (n = 17)	
		Average (Min-Max)	
Length (mm)	Body (from the most forward point of the frons to end of Cauda)	1.98	(1.96-2.39)
	Whole Ant.	1.17	(1.14-1.41)
	Ant. I	0.07	(0.07-0.08)
	Ant. II	0.05	(0.05-0.06)
	Ant. III	0.31	(0.30-0.37)
	Ant. IV	0.17	(0.17-0.20)
	Ant. V	0.14	(0.13-0.16)
	Ant. VIb	0.10	(0.09-0.13)
	PT	0.30	(0.30-0.39)
	URS	0.11	(0.11-0.12)
	Hind femur	0.51	(0.51-0.52)
	Hind tibia	0.69	(0.68-0.70)
	2HT	0.12	(0.11-0.13)
	SIPH	0.04	(0.04-0.05)
	Knob of Cauda	0.06	(0.05-0.06)
	Width of the knob of Cauda	0.06	(0.05-0.06)
	Longest seta on Ant. III	0.06	(0.06-0.07)
	Longest seta on AbdT. III	0.13	(0.12-0.14)
No. of setae on	Mandibular lamina	8	(7-10)
	Ant. I	5	(4-5)
	Ant. II	4	(4-5)
	Ant. III	15	(12-17)
	URS (subsidiary)	8	(7-8)
	Tergite VI between SIPH	10	(9-12)
	Tergite VIII	7	(7-8)
	Median of GP	10	(9-14)
	Posterior margin of GP	19	(17-21)
	Knob of Cauda	8	(7-8)
No. of secondary rhinaria on	Ant. III	0	
	Ant. IV	0	
	Ant. V	0	
Ratio (times)	Whole Ant. / Body	0.59	(0.59-0.61)
	PT / Ant. VIb	3.01	(3.00-3.10)
	PT / Ant. III	0.98	(0.98-0.98)
	URS / 2HT	0.97	(0.93-1.01)
	URS / Ant. VIb	1.17	(1.15-1.18)
	SIPH / Body	0.02	(0.02-0.03)
	SIPH / Ant. III	0.16	(0.15-0.17)
	SIPH / Hind femur	0.10	(0.09-0.10)
	SIPH / Knob of Cauda	0.80	(0.78-0.87)
	Knob of Cauda / Width of the knob of Cauda	1.02	(1.00-1.04)
	Longest seta on Ant. III / Ant. IIIBD	2.62	(2.60-2.73)
	Longest seta on AbdT. III / Ant. IIIBD	5.15	(5.14-5.27)

**Table 3.** Biometric data of *Chaitophorussalijaponicus*.

Part		Apterous vivipara (n = 20)	
		Average (Min-Max)	
Length (mm)	Body (from the most forward point of the frons to end of Cauda)	1.52	(1.37-1.69)
	Whole Ant.	0.83	(0.75-0.91)
	Ant. I	0.05	(0.04-0.06)
	Ant. II	0.05	(0.04-0.05)
	Ant. III	0.17	(0.14-0.19)
	Ant. IV	0.12	(0.10-0.13)
	Ant. V	0.10	(0.09-0.11)
	Ant. VIb	0.09	(0.08-0.10)
	PT	0.23	(0.21-0.24)
	URS	0.08	(0.08-0.09)
	Hind femur	0.31	(0.28-0.33)
	Hind tibia	0.49	(0.45-0.51)
	2HT	0.12	(0.11-0.12)
	SIPH	0.05	(0.05-0.06)
	Knob of Cauda	0.03	(0.03-0.03)
	Width of the knob of Cauda	0.04	(0.04-0.04)
	Longest seta on Ant. III	0.07	(0.05-0.10)
	Longest seta on AbdT. III	0.15	(0.12-0.18)
No. of setae on	Mandibular lamina	8	(7-8)
	Ant. I	6	(5-6)
	Ant. II	4	(4-5)
	Ant. III	7	(5-8)
	URS (subsidiary)	8	(7-9)
	Tergite VI between SIPH	12	(10-15)
	Tergite VIII	9	(8-10)
	Median of GP	5	(4-7)
	Posterior margin of GP	15	(14-18)
	Knob of Cauda	6	(4-6)
No. of secondary rhinaria on	Ant. III	0	
	Ant. IV	0	
	Ant. V	0	
Ratio (times)	Whole Ant. / Body	0.55	(0.51-0.58)
	PT / Ant. VIb	2.55	(2.49-2.61)
	PT / Ant. III	1.32	(1.30-1.35)
	URS / 2HT	0.68	(0.62-0.71)
	URS / Ant. VIb	0.89	(0.84-0.92)
	SIPH / Body	0.03	(0.03-0.03)
	SIPH / Ant. III	0.29	(0.22-0.35)
	SIPH / Hind femur	0.16	(0.14-0.17)
	SIPH / Knob of Cauda	1.50	(1.47-1.52)
	Knob of Cauda / Width of the knob of Cauda	0.77	(0.75-0.78)
	Longest seta on Ant. III / Ant. IIIBD	3.04	(2.94-3.10)
	Longest seta on AbdT. III / Ant. IIIBD	6.04	(5.87-6.24)

**Table 4.** Biometric data of *Chaitophorustremulae*.

Part		Apterous vivipara (n = 7)	
		Average (Min-Max)	
Length (mm)	Body (from the most forward point of the frons to end of Cauda)	2.20	(1.94-2.34)
	Whole Ant.	1.25	(1.09-1.32)
	Ant. I	0.06	(0.05-0.07)
	Ant. II	0.05	(0.04-0.05)
	Ant. III	0.35	(0.31-0.38)
	Ant. IV	0.20	(0.17-0.21)
	Ant. V	0.16	(0.15-0.17)
	Ant. VIb	0.11	(0.10-0.12)
	PT	0.30	(0.25-0.32)
	URS	0.09	(0.08-0.10)
	Hind femur	0.55	(0.49-0.58)
	Hind tibia	0.69	(0.64-0.74)
	2HT	0.12	(0.11-0.14)
	SIPH	0.06	(0.06-0.07)
	Knob of Cauda	0.06	(0.05-0.06)
	Width of the knob of Cauda	0.06	(0.06-0.06)
	Longest seta on Ant. III	0.08	(0.06-0.09)
	Longest seta on AbdT. III	0.17	(0.15-0.20)
No. of setae on	Mandibular lamina	8	(7-10)
	Ant. I	8	(7-10)
	Ant. II	5	(4-6)
	Ant. III	11	(9-13)
	URS (subsidiary)	8	(7-8)
	Tergite VI between SIPH	16	(15-18)
	Tergite VIII	12	(11-13)
	Median of GP	8	(4-10)
	Posterior margin of GP	16	(14-22)
	Cauda	6	(5-7)
No. of secondary rhinaria on	Ant. III	0	
	Ant. IV	0	
	Ant. V	0	
Ratio (times)	Whole Ant. / Body	0.57	(0.51-0.60)
	PT / Ant. VIb	2.73	(2.59-2.84)
	PT / Ant. III	0.85	(0.76-0.91)
	URS / 2HT	0.72	(0.68-0.75)
	URS / Ant. VIb	0.83	(0.79-0.85)
	SIPH / Body	0.03	(0.03-0.03)
	SIPH / Ant. III	0.18	(0.17-0.19)
	SIPH / Hind femur	0.11	(0.10-0.12)
	SIPH / Knob of Cauda	1.19	(1.18-1.21)
	Knob of Cauda/ Width of the knob of Cauda	0.82	(0.75-0.85)
	Longest seta on Ant. III / Ant. IIIBD	3.00	(2.86-3.14)
	Longest seta on AbdT. III / Ant. IIIBD	6.07	(5.87-6.24)
